# Multiteam Systems in Long Duration Exploration Missions: A Qualitative Analysis of Key Characteristics and Challenges

**DOI:** 10.3389/fpsyg.2022.877509

**Published:** 2022-08-12

**Authors:** Dana C. Verhoeven, William S. Kramer, Marissa L. Shuffler

**Affiliations:** ^1^Department of Health Services Research and Administration, University of Nebraska Medical Center, Nebraska Medical Center, Omaha, NE, United States; ^2^Department of Psychology, University of Nebraska Omaha, Omaha, NE, United States; ^3^Department of Psychology, Clemson University, Clemson, SC, United States

**Keywords:** multiteam system, team, teamwork, extreme environment, team process

## Abstract

Given the unprecedented environment of long duration space exploration (LDSE), success simply cannot occur without the coordinated efforts of multiple teams, both in flight and on the ground. These multiteam systems (MTSs) are needed to achieve the complex and dynamic tasks of spaceflight missions that will be longer and more uncertain than any previously experienced. Accordingly, research is limited in terms of how to best coordinate these teams and their dynamics—and in particular, how to best prepare LDSE teams to work across time and space effectively. To begin to address these critical questions systematically, qualitative data was extracted from a series of ten interviews with experts in spaceflight and long duration analog environments. Using thematic analysis techniques, we identified several consistent themes for affective, behavioral, and cognitive elements of teamwork occurring within and between teams. We examine each of these in detail, to identify the dynamics of what is currently known and where research needs to go to provide guidance for spaceflight organizations as well as others attempting to successfully implement MTSs in novel, complex environments.

## Highlights

- Consistent affective, behavioral, and cognitive elements of teamwork emerge.
- Teamwork processes differentially impact inter- and intra-team dynamics.
- Lack of shared identity between teams is a salient issue for long-duration missions.
- Cross-training component teams could alleviate and prevent between team conflict.
- Multiteam meetings and boundary spanners foster shared cognition between teams.

## Introduction

As work demands have become increasingly complex, organizations are turning toward larger systems comprised of teams, or multiteam systems (MTSs), to accomplish multifaceted tasks in challenging environments (Mathieu et al., 2001). These systems leverage the coordinated efforts of multiple component teams to achieve more than any one team could do alone and are well suited for complex environments, such as spaceflight missions, where expertise and knowledge are distributed across numerous teams. While spaceflight exploration has traditionally required missions under a year in length, future missions to deep space will require unprecedent travel durations that may last for years. Conducting a successful long duration space exploration (LDSE) mission is not the responsibility of the astronaut crew alone. Rather, LDSE requires an intricate interplay of crews and teams, from the design and development of the mission, the launch and maintenance of crew safety, and through the successful return of the flight crew to Earth (Vessey, 2014). Indeed, successful LDSE cannot occur without the use of component teams, both in flight and on the ground, to achieve the challenging and dynamic tasks of spaceflight, particularly in the long-term, communication delayed, and unprecedented environment that surrounds LDSE.

While prevalent in complex environments, MTSs have only recently become a focus of organizational research (Shuffler et al., 2014). For example, laboratory studies of student teams have been designed to examine larger numbers of MTSs in a controlled environment and NASA has designed analog environments (e.g., isolation and confinement analogs, bedrest analog, radiation analog; Dunbar, 2020) to allow for high-fidelity quantitative studies. However, these quantitative approaches pose several limitations, including the *ad hoc* nature of the study teams, where participants tend to have little or no prior experience working together before the study and participant response attrition in longitudinal analog studies. NASA has stated that additional research is needed to optimize *how* the network of teams required for spaceflight work together both *within* (intra-team) and *between* (inter-team) teams of the MTS, particularly in high-risk contexts where the cost of failure could be fatal (see: NASA Team Risk Portfolio- Team Gap 102, 103, and 104; Landon, 2022). Capturing teamwork phenome in lab settings is difficult due to limitations in both research methodology (e.g., lack of validated metrics) and resources (e.g., time required to evaluate team processes both *within* (i.e., team level) and *between* (i.e., MTS level) teams of the MTS). Qualitative approaches, such as interviews, offer an opportunity to provide rich information about the nuances of MTS team functioning, while minimizing participant fatigue from lengthy survey questionaries. Such grounded theory techniques allow researchers to gain unique insights into phenome from those who have experienced them firsthand (Corley, 2015).

Accordingly, the present research serves to expand our knowledge of LDSE MTSs via a series of semi-structured interviews with subject matter experts: individuals who have familiarity with and/or operate in MTSs in spaceflight or analog environments. The guiding research questions for the interviews were 1. *How* do the MTS component teams work together across high risk contexts and 2. What issues might be more challenging for MTSs as we move toward missions requiring more crew autonomy and less frequent communication in LDSE. By leveraging this expertise, we enhance current literature on LDSE by (1) expanding upon what is known of MTSs in spaceflight contexts, (2) providing practical recommendations for successful MTS operations in LDSE, and (3) outlining where future research is needed to optimize spaceflight MTSs. Thus, ensuring that as future missions become longer and more complex, we are prepared to address the new challenges that emerge.

To structure our interview findings, we highlight the inter- and intra-team (i.e., within and between team, respectively) processes and emergent states that were discussed, or the affective and cognitive emergent states and behavioral processes that were identified as critical for mission success (Shuffler et al., 2015). Leveraging these data, we explicate how such processes emerge dynamically, how they may differ based on different MTS attributes, and the unique contextual needs of MTSs in high-risk environments. As such, in the sections that follow, we will define MTSs, examine the challenges facing MTSs in LDSE, detail the interview methodology, and describe the inter- and intra-team processes and emergent states reported by interviewees.

## Defining Multiteam Systems and Spaceflight Challenges

Multiteam systems (MTSs) are defined as “two or more teams that interface directly and interdependently in response to environmental contingencies toward the accomplishment of collective goals” (Mathieu et al., 2001, p. 290). The level of analysis for multiteam systems is often considered the meso-level, where effort is orchestrated at a level of analysis higher than the team, but lower than the organization, and potentially span the boundaries of multiple organizations (Mathieu et al., 2001). MTSs exist in variety of contexts and describe networks of teams working toward at least one shared goal, in addition to individual team goals. Such interdependent tasks create challenging situations as they require coordination for multiple, previously unacquainted, teams. Further, MTSs demand that individuals with differing skill sets and specialties be brought together to tackle novel challenges (Goodwin et al., 2012; Lanaj et al., 2013).

**FIGURE 1:**
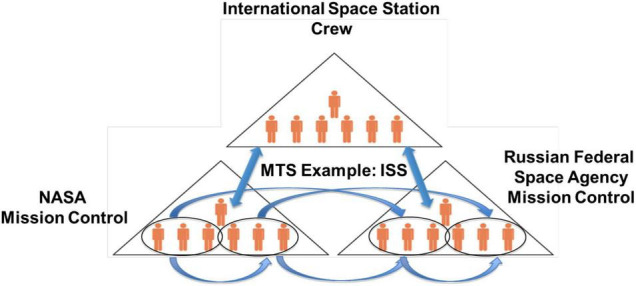
Multiteam systems in spaceflight: international space station example.

In the spaceflight context, multiple MTSs can be observed. For example, the International Space Station (ISS) may be thought of as a long duration MTS, whereby mission controls of different international agencies (e.g., NASA, Russian Federal Space Agency) must work together to ensure the crew is supported during missions (Salas et al., 2015; Mesmer-Magnus et al., 2016). In addition to coordinating with crew members on the ISS, mission controls must also coordinate with one another, and may have additional component teams that coordinate as MTSs to address crew needs, as they arise (See Figure 1). This network of teams form a MTS that must coordinate efforts across multinational, multi-organizational boundaries before, during, and after flight. However, as space exploration moves toward longer duration missions to Mars, the challenges increase exponentially.

Long duration space exploration (LDSE) crews will be multicultural, interdisciplinary, and forced to live and work with the same four to six individuals in a shuttle the size of small kitchen for at least three years (Mesmer-Magnus et al., 2016). In addition to facing interpersonal challenges, crews will have unprecedented autonomy in comparison to prior missions (Mesmer-Magnus et al., 2016). For over 20 years, the ISS has functioned as a multinational, collaborative project, flown by the mission control center (MCC) which monitors mission status in real time, allowing them to immediately address any concerns or issues that arise onboard with numerous teams of ground staff. However, LDSE are inherently more risky than previous missions, as there will be no resupply, reinforcement, or abort capabilities in unforeseen circumstances (Vessey, 2014). Additionally, power and weight requirements dictate that the amount of bandwidth and power consumption available will be significantly lower than current missions, limiting the amount of information that can be transferred between MCC on the ground and the crew. Such restrictions may strain ground-crew relations, creating a divide between frames of reference, thereby resulting in increased goal conflict between teams (Vessey, 2014; Mesmer-Magnus et al., 2016).

Furthermore, as communication becomes increasingly delayed and crews become more autonomous, the structure of the connections between component teams will be affected (Cannon-Bowers et al., 1993). This may lead to challenges for MCC in determining how to respond appropriately, including potentially giving up control over certain tasks or leadership. For example, while historically the MCC has served to manage any unexpected events on the ISS, the estimated 20-min (each way) communication delays that are anticipated during LDSE will reduce the capabilities of MCC (Landon et al., 2017; Larson et al., 2019). Rather than MCC serving as the leader and decision maker of the mission, crews may have to take on leadership roles and leverage MCC as support. This would necessitate the alteration of hierarchical arrangements and temporal orientations, and strain coordination and communication, as goal and leadership structures shift (Zaccaro et al., 2012; Driskell et al., 2018). However, little is known about how these factors might shift, to the success or detriment of the LDSE. Therefore, research is needed to understand how this unique context will shape both team and systemic functioning and identify potential breakdowns to prepare for future missions.

## Materials and Methods

### Sample

To assess how spaceflight teams combine their efforts, 10 subject matter experts (SMEs), comprised of both current and former NASA personnel, were interviewed. To ensure we had a holistic view of the system, interview coordinators from NASA facilitated this process and assisted in garnering SME volunteers to participate. Several different departments within NASA were represented; interviewees had various occupations related to space flight or analogs of extreme environments, including former astronauts, MCC members, and analog participants. Within MCC, we were able to evaluate MTS interactions from the perspective of a flight director, capsule communicator (CAPCOMs), and flight controllers. Together, these interviews provided rich insight of the MTS functioning expected to occur during LDSE by highlighting factors that may enhance or impede effective coordination within and between teams.

### Procedure

Interviews were conducted via conference calls and followed a semi-structured protocol that allowed participants to switch between topics. At the start of each interview, participants were briefed on the purpose of the call and our primary aims. Specifically, participants were told that this effort sought to examine issues regarding the multiple teams that must work together during a spaceflight mission and were asked to leverage their own experiences to discuss specific issues that arise between or across teams. To maintain anonymity, interviews were not recorded and names were omitted from the final report. Instead, research assistants took detailed notes during the conference call to depict participant responses for subsequent thematic analyses.

### Analyses

A qualitative, inductive approach to theory development was used to identify different themes that emerged. In the first phase of this process, two independent coders (authors DV and WK) reviewed the interview notes and current literature on MTSs and created a comprehensive list of affective, behavioral, and cognitive variables critical to MTS functioning. While evaluating such variables is not new in the teams literature, their development and interaction at the MTS level (e.g., meso-level) is functionally distinct from teams theory, given that multiple, distinct teams comprise a MTS, and has received little research (Shuffler et al., 2015).

After identifying these three overarching characteristics and the respective constructs that were exemplars of each, the coders reviewed the interview notes a second time, highlighting passages representing the different sub-constructs (e.g., Theme: affect, construct: identity) and whether the passage identified interactions occurring within (e.g., cohesion within the crew) or between teams (e.g., cohesion between the crew and MCC). The coded passages were then sorted into a final document listing each theme, respective variables, and a final count of how many interviewees mentioned each.

## Results

A summary of the overarching themes and critical competencies for MTS functioning are depicted in Table 1 and summarized in the following sections. Given that each of these constructs can impact both within- and between-team functioning, we also describe the nature and emergence of these variables across organizational levels. Indeed, while some SMEs referenced how a specific factor might impact individual team functioning, other participants noted its criticality *between* teams. The results are organized by the behavioral processes and emergent affective and cognitive states that encompass the highlighted competencies.

**TABLE 1:** Number of interviewees that identified affective, behavioral, and cognitive themes.

Theme	*N*
**Affect**	
Identity	10
Cohesion	6
Trust	3
Psychological Safety	3
Mood	1
Collective Efficacy	1
**Behavior**	
Coordination	6
Conflict	6
Communication	5
Leadership	9
Feedback	3
**Cognition**	
Shared Mental Models (SMM)	8
Transactive Memory System (TMS)	2

*There were N = 10 respondents.*

### Affect

Team affective states are the emotion-related aspects of teams including attitudes toward the team and tasks (Menges and Kilduff, 2015; Bell et al., 2018). Examining affect is not only important in the spaceflight context due to confined quarters and a high risk environment, but it is found to have different effects on MTSs than teams. For example, unlike traditional teams, having a strong, shared intra-team identity might distance a component team from inter-team goals and identity, thereby causing a misalignment from the system’s overarching purpose (Connaughton et al., 2012). Additionally, at the team level, having all team members exhibiting cohesion and trust with one another can have a positive impact on team effectiveness; however, at an MTS level, it is not realistic for every member of the system to feel cohesive with or trust every other member (DiRosa, 2014). Instead, for MTSs, constructs such as trust and cohesion may be most important among key boundary spanners or leaders, thus creating different implications regarding how these constructs should be assessed in regards to their impact on MTS outcomes (Burke et al., 2014; Carter and DeChurch, 2014). To tease apart how affect can impact spaceflight MTSs, we present three affective themes that emerged from our interviews: identity, cohesion, and trust/psychological safety.

#### Shared Identity

Grounded in social identity theory, shared identity emerges via social and group identification (Connaughton et al., 2012). During this emergent process, individuals on a team begin to see themselves as part of an in-group and define how they see themselves using others on the team as a social referent, thereby increasing the commonalities perceived across teammates (Ashforth and Mael, 1989). The idea that it is important for individuals in an MTS to have a shared identity emerged as a consistent theme affecting both teams within MTSs and the MTS as a whole. Indeed, 100% of the interviewees explained that having component teams with different identities can lead to problems for the MTS. For instance, issues discussed ranged from inter-organizational (e.g., “Russian, French, etc. individuals are great but, when you have to work with the organizations, that’s when we get frustrated”) to system-wide problems (e.g., “headquarters didn’t really care about us or respect our wishes”). However, regardless of the foci of the identity misalignments, examples often resulted in an “us versus them” climate where, instead of working together, component teams would be critical of one another (e.g., “ground [mission control] is a collective monster that is not sensitive to our needs”). Such misalignments can have negative repercussions for the MTS, seeing as it is imperative that each component team is comprised of individuals who understand and strive to achieve both team *and* MTS goals (Mathieu et al., 2001).

At the intra-team level, most comments were focused on the idea that, within component teams, it is difficult to build and maintain a shared identity. For instance, when considering the fact that some component teams comprising a spaceflight MTS are interdisciplinary, there can be less interdependence and more individual work. This, in turn, can cause a component team to easily forget their overarching goals and lead to problems in performance (e.g., “we had no unifying goals; we all had our independent goals. We weren’t really one group”). However, in LDSE the crew will likely have increased interdependence given the confined context and the additional responsibilities they will have in comparison to prior missions (e.g., navigating the shuttle autonomously) as they move farther from Earth. As such, one interviewee suggested the crew spend as much time together as possible prior to the mission, to develop an intra-team identity that may then foster inter-team identity.

Finally, comments identified the importance of different teams and individuals to have prior experience with those on their component team and the other teams in the system, particularly when the individuals or organizations they were working with were culturally diverse or had differing protocols and procedures. Without such knowledge, the MTS risks task conflict from the differing protocols between partners. Therefore, if one organization is used to doing things in a very standardized fashion, but the other sees procedures as more dynamic, disagreements regarding the importance of goals were likely to emerge (e.g., “We should be getting better at understanding why the partner is asking for something based on organizational differences in required tasks”).

#### Cohesion

Considering that high levels of cohesion are representative of an individual’s positive relationships with others in their group or MTS and their commitment to goals (Zaccaro, 1991), it is understandable that within a system of teams, cohesion might be more difficult to build and maintain. This is perfectly aligned with our findings that, when cohesion was discussed within teams, examples were positive in nature. However, interviewees consistently brought up negative experiences regarding the lack of between-team cohesion. As such, cohesion was most salient to our interviewees when it was absent (e.g., “away team perceives mission support as asking too much of them [and] mission support doesn’t understand what the crew is going through”). As a construct, cohesion is heavily tied to shared identity in that a lack of cohesion between teams can build a rift and promote an “us vs. them” mentality (Connaughton et al., 2012). For these reasons, some interviewees described issues with “competing objectives” or misaligned perceptions.

Conversely, all three of the instances in which cohesion was discussed in the intra-team context saw alignment on both social relationships and the task, including discussions of there being “no real competition within the crew” and they are “working together for the same goal.” Despite the fact that in traditional team research this is presented as a positive affect for teams, when considered in the MTS context, high levels of cohesion within component teams paired with low levels of cohesion between teams can result in countervailing forces that lead to negative outcomes for the MTS (DeChurch and Zaccaro, 2010). For instance, if a component team shares very strong cohesion but has lost sight of what the other teams do and the overarching MTS goals, there will likely be a lack of information sharing between teams that support gaps in identity. For these reasons, interviewees suggested that pre-flight time for LDSE MTSs be dedicated to building relationships within and across teams to encourage system cohesion.

#### Trust and Psychological Safety

As constructs, these two are very closely tied (Newman et al., 2017). Typically, you find that when a team is willing to trust its team members on personal and task levels, they are more likely to have a climate of psychological safety where they are not afraid to take risks or challenge the status quo out of fear that they will be judged or undercut (Carmeli and Gittell, 2009). As such, interviewees discussed that the presence or lack of inter- and intra-team trust and psychological safety in spaceflight MTSs had a meaningful impact on the success of the individuals, component teams, and system. For instance, 30% of interviewees mentioned trust as an important consideration in LDSE. Their comments were related to the distrust felt toward other teams, resulting in some individuals not providing information or improperly blaming another team for a mistake. Conversely, a few interviewees described examples where they experienced trust (e.g., they “might not agree with the decision but they know who made the call so it worked out”) and offered suggestions to improve trust within and between component teams (e.g., “getting to know who the people watching them are would help by putting a face to a name”).

Interestingly, psychological safety was not mentioned as an issue to consider within component teams. Instead, it was discussed as an inter-team consideration. The main reason cited for this issue was due to an extremely formal hierarchical structure that did not warrant open discussion and communication between teams (e.g., “having a very hierarchical structure doesn’t promote easy dissent to management opinion, has been issue with 2 major accidents”). Additionally, due to the high-risk nature of the task, certain individuals might not bring up issues or concerns out of fear of being reprimanded or removed from the team entirely (e.g., “issues with astronauts not telling someone they are hurt because they don’t want to be pulled from the mission”). For obvious reasons, while it is not possible to remove a person from the space flight team in the middle of their mission, withholding such information from MCC can result in negative repercussions for the entire MTS and, as such, ways to increase trust and build psychological safety should be considered pre-flight.

#### Practical Recommendations and Future Research

Based on all the interviews, our three practical recommendations for promoting affective emergent states (See Table 2) are grounded in creating a shared understanding of procedures and personalization between teams. For instance, by training all component teams on the shared goals of the MTS and building relationships between teams, there is an increased likelihood for a stronger identification with the MTS, not just one’s team (Burke et al., 2014). Furthermore, by creating a set of standardized procedures for introducing new ideas or reporting errors that occur, component teams will be more likely to communicate this information and feel a sense of psychological safety between teams (Weaver et al., 2014). However, to best approach these recommendations and build specific training and procedural guidelines, future research must be conducted to determine how these MTSs work together and where the gaps are between component teams.

**TABLE 2:** Practical recommendations identified in operational assessments relevant to spaceflight MTSs.

**Affective emergent states**
• Utilize training to develop a sense of shared identity with: (1) those on their component team, and (2) those on different component teams. • Impose a standardized system for reporting errors, and reinforce that information sharing is a sign of trust and cohesion. • Utilizing pre-training and socialization, build a climate of psychological safety so that component teams feel comfortable with reporting problems.
**Behavioral processes**
• Train flight directors and psychological support teams to have the KSAOs necessary to serve as boundary spanners for communication and coordination between astronaut crews and ground control teams. • Create communication protocols to limit opportunities for miscommunication and delay in communication can provide autonomy. • Coordinate inter-and intra-team efforts, particularly in relation to shared goals and problem solving. • Leadership will need to come from both ground and astronaut crews—shared leadership is optimal but challenging.
**Cognitive emergent states**
• Generate a clear goal hierarchy amongst leadership to establish shared mental models within and between teams to prevent teams from different organizations or with different functions to purport that their unique goals are more important. • Host regular check-ins with updates across all component teams to establish and maintain accurate, system-wide shared mental models and transactive memory systems. Given the global nature of spaceflight MTSs, these could be completed virtually through short summary emails or message boards on a secure server. • Encourage cross-training of individual team members to streamline information sharing to facilitate transactive memory systems (e.g., shared understanding of who knows what).

### Behavior

Of all the emergent states and processes that occur within MTSs, behaviors are perhaps the most understood. From a behavioral process perspective, coordination, communication, leadership, and more broadly, the action and transition processes described by Marks et al. (2005) have all been studied in the MTS context, with differential effects being found for the inter- and intra-team levels (Lanaj et al., 2013; Murase et al., 2014). However, the majority of behaviors brought up in the interviews focused on between team interactions and the pitfalls that occur as inter-team processes begin to breakdown. The following sections will review the behavioral processes critical for spaceflight MTSs as identified by interviewees, including: leadership, coordination, communication, conflict, and feedback.

#### Leadership

The role of leadership within spaceflight was highlighted by 90% of interviewees. In line with previous research on leadership in MTSs, many expressed a need for shared leadership within LDSE teams to support and compliment the formal leadership structure for ideal system outcomes (Bienefeld and Grote, 2014; Carter and DeChurch, 2014). Indeed, LDSE missions will likely require that leadership is a collective effort whereby both component team and system level leadership exists and comes from multiple sources as opposed to a single leader that manages the entire system (Friedrich et al., 2009). Further, members of MTSs may shift back and forth between leading and following the lead of others, depending on system needs and environmental factors [e.g., “part of good leadership is understanding when you’re looking up and when you’re looking down” (because being a good follower) “is part of being a good leader”]. However, interviewees noted that the current culture may not support such dynamic leadership structures (e.g., “culture is very directive, not much upward communication, very autocratic”), suggesting that a change may be required for future missions.

While many of the interviewees reflected on the role of leadership between teams, 30% noted the importance of leadership within teams, with each of those interviewees emphasizing that shared leadership is often required when making decisions. They highlighted that leaders must strike a balance between serving as an authoritative leader and gathering team input/consensus during decision making to ensure that team members have a voice and avoid wasting too much time. Specifically, one interviewee noted that their leader “put a huge emphasis on consensus” to the point where it was “almost an obligatory consensus.” Thereby irritating crew members because they wanted a decision to be made. In contrast, another reported that the project lead “would make decisions without consulting people” or justifying decisions, resulting in “a lot of stress and some conflict” for other team members. These examples illustrate that rather than forcing consensus or not seeking input from crew members, formal leaders must leverage the input and expertise of others to obtain the necessary information for a final decision.

#### Coordination

The importance of coordinating efforts between teams during spaceflight was a recurrent theme across interviews, as mission success is contingent upon the succinct efforts of multiple teams on the ground, across numerous mission controls, and the crew. Formally defined, coordination is a dynamic process whereby teams “orchestrate the sequence and timing of interdependent actions” (Marks et al., 2001, p. 363). As explained by one interviewee, the nature of LDSE is “truly a systems approach” with expertise and knowledge distributed across the system, making it impossible to solve complex problems without the combined efforts of multiple mission control teams. Failing to leverage this systems approach will cause stakeholders to “miss how to best coordinate an effective outcome,” thus highlighting that effective coordination within and between teams is critical for future missions.

However, coordinating across multiple ground crews and teams may be easier said than done, as 60% of interviewees revealed that one of challenges they faced in past missions stemmed from coordination issues between teams. For example, crew members reported feeling like they were the “integrating force” between the different teams on the ground and contrasting space agencies, as many times the crew was left to disentangle the conflicting goals and messages from the ground. Indeed, all instances of coordination in the interviews reflected its importance due to the intricate, interconnected network of teams required to manage spaceflight missions and the numerous opportunities present for disconnects between them. One interviewee clearly illustrated this issue, stating that the problem “is getting organized and staying organized across the ground controls. If you can do that, you can take the stress off the crew. If you’re poor at that, it’s up the crew to resolve the issues in regards to conflicts in tasks or priorities.” This becomes increasingly more complicated for the crew as you consider the different agencies, cultures, and priorities they are tasked to coordinate between. In future LDSE, relying on the crew to serve as the key boundary spanner between teams may not be possible due to inevitable communication delays (Vessey, 2014). Therefore, much more effort and initiative between ground controls must be taken both pre-flight and during flight to, not only create a unified plan and goal hierarchy for the crew as they go into their mission, but also ensure that the different agencies’ goals and priorities are considered when making decisions and communicating with the crew throughout the mission. These coordination processes should be considered early in the flight preparation process, and steps should be taken to put appropriate checks and balances in place to ensure each mission control is being included throughout the mission.

#### Communication

The role of communication in future LDSE has received considerable attention due to the expected communication delays that will occur between the crew and ground as the crew gets farther from Earth, making it a common theme among interviewees, with 50% noting its importance. As previously discussed, astronaut crews will have more autonomy as communication processes become more delayed than previous missions, which interviewees believe will contribute to increases in disconnects and miscommunications (e.g., “We expect there to be a greater number of disconnects and misunderstandings between ground and crew and the same sort of thing between crew and family and the communication across groups.”). These inter-team issues were recurrent, in which all references to communication in the interviews denoted the importance of between team interactions in LDSE, often citing negative outcomes as a result of communication breakdowns. Indeed, instances of conflict due to miscommunication or a lack thereof were echoed across interviews, acknowledging the importance of training “both the crew and mission control to … raise a yellow flag and acknowledge the disconnect between the two” as issues arise, to mitigate breakdowns before they become detrimental to MTS effectiveness.

In addition to discussing the role that communication delays may play in MTS functioning, interviewees also highlighted that teams must appropriately share information across the system by identifying what content should be shared with specific teams versus the entire system. For example, one interviewee reportedly felt like mission critical information was withheld from them while onboard the ISS because they were not told the significance or purpose of their research efforts (e.g., “significance of what was being done was not explained to them well”). It is important that everyone is “on the same page,” particularly between teams and agencies (e.g., NASA and Russia), as both miscommunications and withheld information may lead to detriments in system cognition, creating misalignment between teams and subsequent breakdowns in team performance (Mesmer-Magnus and Dechurch, 2009). Therefore, clear communication protocols should be implemented that determine appropriate teams to incorporate in communications and the best method or modality (e.g., electronic, face-to-face, mixed) for transmitting specific kinds of information between teams to ensure information is being transmitted, received, and understood across the system.

#### Conflict

Particularly in LDSE MTSs, there are many opportunities for conflict, as evidenced in 60% of our interviews. While some instances of conflict revolved around competing task objectives, others cited other affective, behavioral, or cognitive issues. The primary instances of conflict stemmed from competing objectives between different agencies (e.g., NASA vs. Russia), differences in functional expertise (e.g., scientist vs. engineer), and contrasting MCC and crew perspectives. Such conflicting viewpoints often resulted from a lack of shared identity between component teams, which subsequently created system misalignment, facilitating task conflict among members. Indeed, the contrasting perspectives of different component teams led to tension surrounding goal priority and problem-solving approaches. For example, the conflicting viewpoints of the crew and ground was best illustrated when an interviewee reflected on their contrasting approaches to solving a problem onboard. In this instance, the crew asked if they could use a valuable resource onboard to solve a problem. However, the ground “didn’t want to take the risk” of the using the resource when it was not necessary. This example highlights an “idea where the crew was looking tactically at an issue”, while the “ground was looking at it strategically.” Although the crew felt they successfully identified a solution, the MCC may take a more holistic approach to problem solving and prioritize resource preservation.

Differences in crew and ground frames of reference were also shown to generate task conflict when interviewees reported instances where the crew “did not agree with some of the tasking” that the ground had outlined and inquired about changing or cancelling the task. Typically, the crews’ perspectives were cast aside as they were told “Nope. Follow the timeline but thanks for the ideas.” Indeed, one interviewee reported that they were only able to change their task after they all decided to “band against it” and “used all channels to ask” to alter the predetermined tasking timeline. These examples highlight that the crew and ground may have conflicting standpoints toward taskwork and goal priorities, which will play a pivotal role in future LDSE as crews gain more autonomy and interact with MCC less frequently. Therefore, to avoid exceedingly strained relationships between different component teams, steps should be taken to ensure that teams possess a similar perspective and unified understanding regarding mission priorities in an effort proactively mitigate the risk of future conflict.

#### Feedback

Understanding what the system is doing well or may need to improve upon is an integral component to training effectiveness that ought to be incorporated throughout all phases of the mission and mission preparations. Feedback provides individuals, teams, and the MTS as a whole with information on what needs improvement or what they are doing well. Interviewees noted the importance of prompt and timely “feedback for all team members” to improve learning and transfer of the training content, thereby increasing its effectiveness. Overall, 50% of interviewees cited feedback as a critical component of mission success, emphasizing the need for individually tailored, rather than team or system level, feedback. Indeed, feedback is “extremely important after training” and the crew appreciated receiving “specific, individual feedback on research” to ensure their data collection efforts were valuably contributing to the system and future spaceflight improvements.

While feedback is critical to foster development within and between teams, interviewees reported limited opportunities for developmental feedback due to the present culture surrounding training initiatives. The severe consequences associated with failure in LDSE has fostered a training environment that is “very success-oriented” where error avoidance is the prominent practice among trainees, and trainers seem “reluctant to point out mistakes or point out fault” in fear of discouraging trainees. This perspective is also evident during performance appraisals, as team members expressed that “you never get what you need to hear and what you need to change.” Similar results have been found in diary studies where astronauts detailed their frustration with the lack of corrective feedback (Stuster, 2016; Goemaere et al., 2019). Therefore, more honest and complete assessments of individuals, teams, and MTS functioning as a whole are necessary to ensure effective operations and to provide the opportunity to improve, which was noted as one of the “most positive” things that could be done in terms of training. However, it is also worth noting that feedback may appear limited because trainees were already highly functioning. As such, ensuring that there are standardized practices for trainers to evaluate training and engaging trainees in debrief exercises will help mitigate these challenges to ensure trainings objectives are met. Moreover, trainers might benefit from additional training related to providing feedback to the crew in an effort to ensure standardization.

#### Practical Recommendations and Future Research

The aforementioned behaviors highlight the varying different actions that can facilitate or hinder effectiveness across the system in LDSE. Indeed, differences in preferences for leadership structures and decision-making processes were noted as a point of conflict when working with other agencies, as were differences in established behavioral norms for different processes, such as communication and coordination, across organizations and cultures. Considering these processes together, we offer training recommendations (See Table 2) that aim to foster shared leadership across the system to better address the shifting nature of leadership and power that will likely emerge in LDSE. In addition, it would also be beneficial to create communication and coordination procedures that span across the system and respective agencies to foster clear expectations and information sharing within the MTS. Future research should leverage network approaches to assess and capture the more compilational nature of MTS processes and emergent states, whereby such higher-level variables are not the same in their makeup as at lower levels (e.g., Bell et al., 2018; Brown et al., 2021). These approaches would help in understanding the behavioral patterns that emerge at the group level, providing not only a summary of what might be occurring in a MTS, but a direct snapshot of the state of a system at a given point in time.

### Cognition

Team cognition often refers to the shared understanding that team members have of their work, roles, and approach to completing tasks (DeChurch and Mesmer-Magnus, 2010). Constructs such as shared mental models (SMMs) and transactive memory systems (TMSs) assess these aspects of shared cognition and play a critical role in coordinating efforts between the crew and ground for spaceflight (Dechurch and Mesmer-Magnus, 2015). Indeed, NASA has already recognized the value of shared mental models for astronaut crew functioning, with a recent review revealing that shifts in shared mental models that are likely to occur during LDSE (DeChurch and Mesmer-Magnus, 2010). While such shifts may be inevitable, MTSs face many challenges in terms of coordinating, building, and maintaining shared mental models at *both* inter- and intra-team levels (Larsen et al., 2014), as the development of cognitive structures at only one level may impede functioning at the other level. For example, if component teams develop only internal TMS instead of developing a system level TMS, teams may struggle with identifying as part of the larger system and fail to coordinate with other teams to leverage everyone’s expertise (Burke et al., 2003, 2014). However, little research has considered what cognitive structures would be best in the MTS LDSE context. Therefore, the following sections examine the role and emergence of cognition as members coordinate within and across multiple teams in spaceflight.

#### Shared Mental Models

A key factor in future LDSE success relies on the MTS establishing accurate and shared mental models of mission status, expectations, and goals throughout the mission. Overall, 80% of interviewees emphasized the importance of sharing information within and across teams, with 40% noting the importance of team-focused mental models (e.g., interpersonal interaction requirements; Mohammed et al., 2000) and 80% highlighting the role of task-focused mental models [e.g., work goals and performance requirements; (Cannon-Bowers et al., 1993)]. Overall, the majority of within-team comments pertained to the importance of the crew knowing and understanding their team members and knowing how to “react to each person’s needs” to provide support during confinement. In contrast, when considering the larger system, the importance of maintaining a shared understanding of goals and the current mission status across teams was a recurrent theme (e.g., “before you launch something like this you have to have everyone on the same page with the same large goals.”).

The importance of SMM in high-risk situations was also highlighted by interviewees, noting the criticality of both the ground and crew maintaining a shared understanding of the system and shuttle operations to ensure the crew would be equipped to respond to crisis situations (e.g., equipment malfunctions) autonomously if needed. While there were some comments regarding improvements to current procedures [e.g., “team meetings should be done more, so each team understands one another” (to) “reduce mixed messages via shared goals”], most commented on the importance of sharing goals and expectations. However, the conflicting structures, priorities, and procedures that exist between organizations impede this process and often result in misalignment and conflicting goal priorities between teams. To alleviate this issue, it would be helpful to allow crew members to get to “know one another beforehand” to understand “where the other partner is coming from.” Further, one crew member suggested the importance boundary spanners, where someone would be designated to “move between” the different groups involved in the mission to “keep them communicating” and ensure they maintained a shared understanding of the environment.

#### Transactive Memory System

This component of team cognition refers to the shared understanding of what team members and/or component teams possess specific sets of information and knowledge and was discussed by 20% of the interviewees (Peltokorpi, 2008). When TMSs are effective, team members can easily assess who should be responsible for which task based on a mutual understanding of expertise (Lewis and Herndon, 2015). However, as MTSs gain component teams and members, it becomes increasingly more challenging to create a unified TMS across the system. Rather, component teams create TMSs with those they work most closely, as members’ perspectives are influenced by their individual positions within the spaceflight MTS. For instance, during the interviews, flight directors recognized the necessity of the multitude of component teams for mission success, whereas behavioral health and performance personnel focused only on the component teams necessary to ensure the mental and physical health of the space crew. The importance of transactive memory systems were discussed across true and analog space missions, with one interviewee commenting that “often two teams will think that they have overlapping responsibilities and capabilities when there’s actually a gap because people are trying to be respectful. If we both turn away because we think it’s the other person’s job, [that] is when bad things happen.” Establishing and maintaining a shared understanding of the knowledge that different members within the system possess and clear roles and expectations can negate the issue of tasks “falling between the cracks,” particularly in such high-risk environments where teams are highly interdependent.

However, in spaceflight MTSs these networks of knowledge can be very complex and detailed, given the amount of functional diversity that is prevalent across the system. Therefore, understanding how to establish such system level transactive memory systems as well as the factors that promote or inhibit their development is a critical practical and research issue. In response to the challenges of establishing transactive memory systems for LDSE MTSs, one interviewee suggested improving the crew’s understanding of the space craft’s systems to allow them to address issues during a mission without complete reliance on ground control, which also emphasizes the importance of autonomy in future missions.

#### Practical Recommendations and Future Research

The aforementioned, anecdotal evidence reinforces the fact that shared mental models and transactive memory systems are particularly important for MTS functioning. Interviewees repeatedly noted the importance of having a shared understanding across the system in terms of both goals and where information is stored. However, within these systems differences in interdependence across component teams can affect the degree to which they require more complex, shared cognitive/attitudinal structures. While higher levels of interdependence between component teams may result in a need for stronger cognitive (e.g., shared mental models) and affective (e.g., trust) states in order to enable effective performance, this may not be as critical in less interdependent teams (Rico et al., 2018). Therefore, our three practical recommendations provide actionable opportunities for spaceflights MTSs to leverage cross-training and system-wide meetings to facilitate shared cognition amongst interconnected teams within the system (see Table 2). However, future research is needed to better understand the temporal dynamics of cognition in teams and MTSs, as many researchers have called for a better understanding of how both teams and MTSs change and develop as a function of time (e.g., Shuffler et al., 2015), which is particularly critical for understanding how MTSs may play a role in LDSE.

## Discussion and Conclusion

While previous studies have examined the impact of confinement on humans (e.g., Morphew, 2001; Pagel and Choukèr, 2016), individual differences to consider in the selection of LDSE crew members to optimize teamwork (e.g., Landon et al., 2017), and the challenges that individual teams would face in LDSE (e.g., Roma and Bedwell, 2017), we extend this research by examining the practical challenges faced by chosen team members in maintaining effective within and between team processes. Indeed, the themes and recommendations speak to the multitude of complexities that are faced. A LDSE mission will require a multi-national MTS with individuals from different cultures, agencies, and specialties creating a complex network of goal priorities and competing demands. The competing demands stem from the multiple different memberships (i.e., multimembership; see: O’Leary et al., 2012) of crew members, where each group (e.g., culture, agency, profession/specialty) possesses a different priority that the MTS must balance.

Using semi-structured interviews, this paper disentangles some of the aforementioned challenges by identifying the affective states, behaviors, and cognitions identified as imperative for mission success. Further, in our findings, we uncover novel perspectives centered on our two focal research questions (i.e., describing MTS intra- and inter-team processes in high-risk contexts, and the challenges that emerge with increased crew autonomy) and answer the calls of past researchers for the detailed examination of the challenges that emerge for MTSs in LDSE (e.g., Mesmer-Magnus et al., 2016; Landon et al., 2018). Additionally, it addresses the concern highlighted in previous research by Zaccaro et al. (2020) who found that only 7.8% of all existing papers on MTSs have component teams that are intellectually/functionally diverse. As there is a strong variance in the knowledge and specialties across component teams in LDSE, we shed light on novel challenges faced by these MTSs and help build upon an understudied topic.

### Theoretical and Practical Implications

Across all research on MTSs (e.g., Shuffler et al., 2015), including that which is specific to spaceflight (e.g., Dechurch and Mesmer-Magnus, 2015), there are calls for a more nuanced understanding of how cognitions emerge both within- and between-teams. For example, while existing research provides detail as to how shared mental models emerge in a single team, we are still unsure how MTSs integrate multiple shared mental models across component teams (Dechurch and Mesmer-Magnus, 2015). Through our research we shed light on some of these questions and provide initial evidence that there may be a tie between the interdependence of component teams and their need for strong shared mental models. As such, how closely MTS component teams work together could provide us with a targeted approach to resolving communication issues and misalignment in the system via targeted interventions. Future research should be conducted to confirm this finding, as it would draw implications for MTSs across organizations and in spaceflight alike.

Moreover, while our paper focuses on each type of emergent state that occurs within MTSs independently (i.e., affect, behaviors, and cognition), the practical recommendation of using multilevel interventions was universal across all three. Specifically, we propose that the cross-training of interdependent, component teams (e.g., astronaut crew and ground control) will lead to widespread benefits ranging from an increased shared identity to effective communication and information sharing. This is an idea that, while commonplace in the empirical literature surrounding teams (e.g., Marks et al., 2002), is understudied at the MTS level. As such, we propose that future research is conducted to determine the important best practices that should be considered in cross-training component teams.

Lastly, due to the multi-organizational nature and unique specializations expected of future component teams in LDSE MTSs, there is a consistent need for a shared language and understanding across all teams. This finding is supported by the limited previous research that exists on MTSs with component teams in different organizations (e.g., Lavallée and Robillard, 2018). To succeed in the LDSE setting, we suggest that a shared language is achieved across component teams that engage in cross-training. This will reduce ambiguity and confusion across teams that could lead to hesitation in communication and reduced collaboration. Moreover, past research has shown that by simply standardizing debriefings, teams can experience a 25% increase in future performance (Tannenbaum and Cerasoli, 2013). When considering a high-risk context like LDSE, any increase in future performance could lead to lives being saved. These findings were also found to hold consistent over time in the review of existing literature that was conducted by Keiser and Arthur (2021). As there is minimal research on building shared language across MTSs, future research should explore the benefits and challenges that come with this. For instance, while a shared language might align mental models and promote a shared identity across the MTS, would component teams struggle with the integration of this language into their own intra-team communications to the detriment of intra-team shared identity?

### Limitations

Our findings must be considered through the lens of several limitations. First, the findings of this study represent what interviewees anticipate will be the most important aspects of teamwork for teams participating in LDSE, given that there have been no prior deep space missions. Therefore, recommendations are limited to interviewees’ prior experiences on ISS, MCC, and in analog environments. The results of this study are not meant to encompass all aspects of teamwork that might be relevant to LDSE, but reflect the most critical aspects of team affect, behavior, and cognition identified by subject matter experts in the interviews. Therefore, additional aspects of teamwork and leadership might be applicable that were not highlighted in our interviews and, therefore, omitted from our paper. In addition, privacy and anonymity policies limited the depth of the descriptive information provided about the subject pool (e.g., exact counts of professions represented, years of experience). However, all personnel were selected by NASA due to their experiences and expertise to ensure we had a representative group of subject matter experts. Overall, results of the thematic analysis enhance current literature on LDSE by (1) expanding upon what is known of MTSs in LDSE contexts, (2) proving practical recommendations for successful MTS operations, and (3) outlining where future research is needed to optimize spaceflight MTSs.

### Conclusion

In sum, MTSs in spaceflight contexts are presented with many unique challenges caused by their environment. They require networks of teams to coordinate efforts across time and space, placing the optimization of MTS functioning as a central and critical component of LDSE (Vessey, 2014). Our interviews reflect this by showing that consistent themes emerged for affective, behavioral, and cognitive components of teamwork both within and between component teams. By examining each of these in detail, we help identify the dynamics of what is currently known and where research needs to go (See Table 2 for a comprehensive list of recommendations). Finally, we hope that our findings provide guidance and understanding to spaceflight organizations implementing MTSs in these novel, complex environments.

## Data Availability Statement

The original contributions presented in this study are included in the article/supplementary material, further inquiries can be directed to the corresponding author.

## Ethics Statement

Ethical review and approval was not required for the study on human participants in accordance with the local legislation and institutional requirements. Written informed consent for participation was not required for this study in accordance with the national legislation and the institutional requirements.

## Author Contributions

DV lead development of the manuscript, qualitative analyses, transcribed interviews, and wrote sections of the manuscript. WK assisted in writing the manuscript, served as the secondary coder for qualitative analyses, and transcribed interviews. MS was the PI on the grant, conducted interviews, assisted with idea generation, and reviewed the manuscript. All authors contributed to the article and approved the submitted version.

## Author Disclaimer

The views expressed in this work are those of the authors and do not necessarily reflect the organizations with which they are affiliated or their sponsoring institutions or agencies.

## Conflict of Interest

The authors declare that the research was conducted in the absence of any commercial or financial relationships that could be construed as a potential conflict of interest.

## Publisher’s Note

All claims expressed in this article are solely those of the authors and do not necessarily represent those of their affiliated organizations, or those of the publisher, the editors and the reviewers. Any product that may be evaluated in this article, or claim that may be made by its manufacturer, is not guaranteed or endorsed by the publisher.
